# The relationship of the oleic acid level and *ECHDC3* mRNA expression with the extent of coronary lesion

**DOI:** 10.1186/s12944-016-0312-6

**Published:** 2016-09-01

**Authors:** Mychelle Kytchia Rodrigues Nunes Duarte, Jéssica Nayara Góes de Araújo, Victor Hugo Rezende Duarte, Katiene Macêdo de Oliveira, Juliana Marinho de Oliveira, Antonio Augusto Ferreira Carioca, Raul Hernandes Bortolin, Adriana Augusto Rezende, Mario Hiroyuki Hirata, Rosário Domingues Hirata, Dan Linetzky Waitzberg, Severina Carla Vieira Cunha Lima, André Ducati Luchessi, Vivian Nogueira Silbiger

**Affiliations:** 1 Postgraduate Program of Nutrition, Federal University of Rio Grande do Norte, Natal, Brazil; 2 Department of Clinical and Toxicological Analysis, Federal Universty of Rio Grande do Norte, Natal, Brazil. Avenue General Gustavo Cordeiro de Farias, S/N, Natal, Rio Grande do Norte CEP: 59014-520 Brazil; 3 Hospital Universitário Onofre Lopes, Federal University of Rio Grande do Norte, Natal, Brazil; 4 Nutrition Department, School of Public Health, University of São Paulo, São Paulo, Brazil; 5 Faculty of Pharmaceutical Sciences, University of São Paulo, São Paulo, Brazil; 6 Faculty of Medicine (LIM-35), University of São Paulo, São Paulo, Brazil; 7 Department of Nutrition, Federal University of Rio Grande do Norte, Natal, Brazil

**Keywords:** Oleic acid, *ECHDC3*, Friesinger index, Monounsaturated fatty acids, Cardiovascular diseases

## Abstract

**Background:**

The fatty acid profile is associated with the risk and progression of several diseases, probably via mechanisms including its influence on gene expression. We previously reported a correlation between *ECHDC3* upregulation and the severity of acute coronary syndrome. Here, we assessed the relationship of serum fatty acid profile and *ECHDC3* expression with the extent of coronary lesion.

**Methods:**

Fifty-nine individuals aged 30 to 74 years and undergoing elective cinecoronariography for the first time were enrolled in the present study. The extent of coronary lesion was assessed by the Friesinger index and patients were classified as without lesion (*n* = 18), low lesion (*n* = 17), intermediate lesion (*n* = 17) and major lesion (*n* = 7). Serum biochemistry, fatty acid concentration, and *ECHDC3* mRNA expression in blood were evaluated.

**Results:**

Elevated serum levels of oleic acid and total monounsaturated fatty acids were observed in patients with low and intermediate lesion, when compared to patients without lesion (*p* < 0.05). *ECHDC3* mRNA expression was 1.2 fold higher in patients with low lesion than in patients without lesion (*p* = 0.020), and 1.8 fold lower in patients with major lesion patients than in patients with low lesion (*p* = 0.023).

**Conclusion:**

Increased levels of monounsaturated fatty acids, especially oleic acid, and *ECHDC3* upregulation in patients with coronary artery lesion suggests that these are independent factors associated with the initial progression of cardiovascular disease.

## Background

Cardiovascular disease (CVD) is the leading cause of death worldwide. The early diagnosis of CVD along with proper assessment of cardiovascular risk is crucial for further reduction of health care costs and mortality rates. Knowledge of the CVD physiopathology seems to be the best approach to achieve these goals. ‘Omics’ analysis can contribute to this field by providing fundamental information to better understand complex biological systems, such as atherosclerosis [1], the primary origin of most common cardiovascular disorders [2].

Particularly, transcriptomics is a relevant tool for the identification of diagnostic/prognostic biomarkers for CVD. In a microarray-based gene expression study performed previously by our research team, we found that *ECHDC3* mRNA expression was significantly increased within 2 h after an acute coronary syndrome, suggesting this gene could be a potential novel biomarker for the early stage of an acute episode [3]. However, the function of ECHDC3 or its involvement with cardiovascular diseases is scarcely explored in the literature, which might be due to its recent identification. *ECHDC3* encodes enoyl-CoA hydratase domain containing 3, a mitochondrial enzyme that has a crotonase-like domain similar to enoyl-CoA hydratase [4]. Furthermore, ECHDC3 is presumed to be involved in β-oxidation, the most important and well-known pathway for fatty acid (FA) oxidation [5–7].

The consumption of specific dietary FAs has been shown to influence risk and progression of several chronic diseases, such as CVD, possibly by altering gene expression [8, 9]. However, studies in this field have provided ambiguous results, especially regarding the effects of monounsaturated FAs (MUFAs) on risk for coronary heart disease [10]. Since there is no study evaluating the relationship between FA levels and genes related to CVD development and progression yet, we aimed to evaluate the serum FA profile and *ECHDC3* expression in patients with varying extent of coronary lesion.

## Methods

### Study population

Fifty-nine male and female individuals aged between 30 to 74 years that were undergoing cinecoronariography to investigate extent of coronary lesion were enrolled in the present study. Exclusion criteria included diagnosis of cardiomyopathy, heart valve disease, congenital diseases, pericarditis, coronary revascularization, chronic kidney disease, liver failure, endocrine disorder (except for diabetes mellitus), inflammatory diseases, malignant diseases, blood disorders, autoimmune diseases and family history of hypercholesterolemia. Patients were selected at the Hemodynamics unit of Hospital Universitário Onofre Lopes. This study was approved by the hospital’s Research Ethics Committee under protocol number CAAE 0001.0.051.294-11. All participants signed an informed consent form.

### Blood samples and biochemical analysis

Peripheral blood samples were obtained from patients. Fasting serum glucose, total cholesterol and fractions, urea, creatinine K, uric acid, alanine and aspartate aminotransaminases were measured using colorimetric and colorimetric-enzymatic methods in BIO-2000 IL, a semi-automatic biochemical analyzer (Bioplus, São Paulo, Brazil). Values of LDL-cholesterol were calculated according to the Friedewald formula [11].

### Extent of coronary lesion

The extent of coronary lesion was assessed by the Friesinger index [12, 13]. Each of the three main coronary arteries (anterior descending, circumflex and right coronary) was scored separately from zero to five. Subjects were classified as without lesion when the Friesinger index was equal to 0; low lesion, when it varied from 1 to 5; intermediate lesion, 6–10; and major lesion, 11–15, as adapted from Chagas et al. (2013) [14].

### Gas chromatography

Total serum lipids were extracted using a mixture of methanol and chloroform chromatographic solution (2:1, vol/vol), and FAs were converted to FA methyl esters using a modified sodium methoxide method [15]. Then the FA profile was estimated using flame-ionization gas chromatography on a device (CG-2010, SHIMADZU, Kyoto, Japan) equipped with a DB-FFAP capillary column (15 m × 0.100 mm × 0.10 μm [J&W Scientific from Agilent Technologies, Folsom, CA, USA]). Individual peaks were quantified as the area under the peak and results expressed as percentages of the total area of all FA peaks [16].

### RNA extraction and RT-qPCR

Total RNA was extracted from blood samples previously stored in RNAlater® Stabilization Solution (Life Technologies, Carlsbad, CA, USA), using an RiboPure^TM^ – Blood kit (Life Technologies). RNA integrity was assessed by 1 % agarose gel electrophoresis with MOPS buffer, while its quantity was measured using Qubit® 2.0 Fluorometer (Life Technologies). cDNA synthesis was performed using a High Capacity cDNA Reverse Transcription Kit (Applied Biosystems, Foster City, CA, USA) in a MyCycler Thermal Cycler (BIO-RAD, Philadelphia, PA, USA). RT-qPCR was performed using *ECHDC3* TaqMan Assay (Hs00226727_m1) (Life Technologies). The reference gene was selected from a normalization study of three endogenous candidate genes: *GAPDH* (Hs.592355), *ACTB* (Hs.520640), and *18S rRNA* (Hs.626362). RT-qPCR reactions were carried out in 96-well plates using the 7500 Fast Real-time PCR System (Applied Biosystems). Relative expression of *ECHDC3* was calculated using the 2^-deltaCT^ method [17] with *ACTB* as a reference gene.

### Statistical analysis

Statistical analysis was performed using SPSS® 22.0 software (SPSS, Chicago, IL, USA). Normal distribution was evaluated using the Kolmogorov-Smirnov test. Continuous variables with normal distribution are presented as mean and standard deviation and were compared using ANOVA followed by Student’s *t*-test. Variables with skewed distributions are presented as median and interquartile range and were analyzed using the Kruskal-Wallis test followed by Mann-Whitney’s test. Categorical variables were compared by chi-square test. Spearman and Pearson correlations were performed between all continuous variables. The level of statistical significance was accepted as *p* < 0.05.

## Results

According to the Friesinger index, the patients were classified into four groups: without lesion (*n* = 18), low lesion (*n* = 17), intermediate lesion (*n* = 17), and major lesion (*n* = 7). Demographic, anthropometric, clinical, and biochemical data of these groups are shown in Table 1. We did not find significant differences among the groups regarding clinical and biochemical data.

**Table 1:** Demographic, anthropometric and clinical data of patients classified according to the extent of coronary lesion

Variables	Total (*n* = 59)	Without lesion (*n* = 18)	Low lesion (*n* = 17)	Intermediate lesion (*n* = 17)	Major lesion (*n* = 7)	*p-*value
Age, years	60.0 ± 9.0	55.0 ± 9.0	62.0 ± 9.0	63.0 ± 10	60.0 ± 7.0	0.080
Sex male, %	54.2	44.4	58.8	64.7	42.9	0.582
BMI, kg/m^2^	26.82 ± 5.65	27.86 ± 4.70	25.27 ± 8.52	26.92 ± 4.29	27.43 ± 1.13	0.630
Obesity, %	20.3	33.3	17.6	17.6	0	0.133
Dyslipidemia, %	91.5	77.8	100.0	94.1	100.0	0.080
Diabetes mellitus, %	30,5	33.3	23.5	35.3	28.6	0.884
Hypertension, %	79.7	77.8	76.5	82.4	85.7	0.944
Diastolic pressure, mmHg	84.0 ± 20.0	81.0 ± 8.0	80.0 ± 24.0	90.0 ± 18.0	90.0 ± 32.0	0.355
Systolic pressure, mmHg	143.0 ± 26.0	137.0 ± 24.0	139.0 ± 22.0	155.0 ± 32.0	141.0 ± 21.0	0.187
Alcoholism, %	18.6	27.8	17.6	5.9	28.6	0.350
Smoking, %	22.0	27.8	17.6	17.6	28.6	0.825
Physical activity, %	47.5	50.0	58.8	35.3	42.9	0.573
Biochemical Analyses						
Glucose, mmol/l	5.16 (3.72–21.87)	4.75 (3.89–14.26)	4.88 (3.75–11.04)	5.55 (3.72–21.87)	5.94 (4.44–13.04)	0.443
Total cholesterol, mmol/l	4.61 (2.95–7.85)	4.36 (3.08–6.50)	4.51 (3.19–6.89)	5.05 (2.95–7.85)	4.61(3.65–7.02)	0.555
HDL-cholesterol, mmol/l	0.92 (0.41–1.45)	0.95 (0.57–1.45)	0.93 (0.54–1.30)	0.91 (0.41–1.32)	0.83 (0.62–1.40)	0.669
LDL-cholesterol, mmol/l	2.68 (1.21–5.74)	2.38(1.49–4.57)	2.63(1.21–5.00)	3.26 (1.21–5.74)	2.89 (1.93–5.27)	0.419
Triglycerides, mmol/l	1.62 (0.75–9.47)	1.63 (0.79–6.94)	2.25(0.86–6.96)	1.62(0.75–9.47)	1.45 (0.85–3.24)	0.612
ALT, μKat/l	0.43 (0.17–2.19)	0.38 (0.17–1.57)	0.43 (0.25–1.04)	0.33 (0.17–2.19)	0.60 (0.25–1.45)	0.723
AST, μKat/l	0.52 (0.25–1.57)	0.52 (0.25–1.49)	0.43 (0.25–0.95)	0.52 (0.25–1.57)	0.60 (0.25–1.57)	0.546
Ureia, mmol/l	5.85 (3.34–10.35)	5.93 (3.34–9.52)	5.68 (4.31–9.85)	5.93(3.37–10.35)	5.85 (4.84–6.35)	0.693
Creatinine, μmol/l	79.56 (17.68–141.44)	79.56 (53.04–141.44)	79.56(53.04–123.76)	88.40 (53.04–106.08)	70.72 (17.68–106.08)	0.323
Uric acid, mmol/l	0.29 (0.11–0.52)	0.27(0.16–0.52)	0.28 (0.11–0.43)	0.29 (0.11–0.43)	0.33 (0.14–0.37)	0.965

Data are presented as mean ± standard deviation for parametric samples and as median (interquartile range) for non-parametric samples. Categorical variables were compared by Chi-square test. Parametric analysis was performed by ANOVA way. Non-parametric samples were performed by Kruskal-Wallis test. *P*-values < 0.05 were considered statistically significant in all statistical test. *BMI* body mass index, *HDL-cholesterol* high density lipoprotein, *LDL-cholesterol* low density lipoprotein, *AST* aspartate aminotransferase, *ALT* alanine transaminase

FA serum concentrations of the different groups are shown in Table 2. Patients with low and intermediate lesion presented higher concentrations of oleic acid (*p* = 0.025 and *p* = 0.034, respectively) compared to patients without lesion. Similarly, MUFA levels were higher in patients with low and intermediate lesion (*p* = 0.023 and *p* = 0.040, respectively) than in patients without lesion. We did not observe significant changes in other FAs among the groups.

**Table 2:** Serum fatty acid concentration according to the extent of coronary lesion

Fatty acids (%)	Total (*n* = 59)	Without lesion (*n* = 18)	Low lesion (*n* = 17)	Intermediate lesion (*n* = 17)	Major lesion (*n* = 7)	*p-*value
SFA	44.12 ± 3.85	44.70 ± 4.18	43.79 ± 4.46	43.93 ± 2.74	43.93 ± 4.40	0.904
Myristic (C14:0)	0.89 ± 0.30	0.94 ± 0.36	0.91 ± 0.26	0.87 ± 0.31	0.80 ± 0.19	0.756
Palmitic (C16:0)	30.83 ± 3.07	31.40 ± 2.95	30.61 ± 3.15	30.52 ± 2.80	30.63 ± 4.22	0.830
Stearic (C18:0)	12.40 ± 1.89	12.36 ± 2.15	12.27 ± 2.10	12.54 ± 1.70	12.50 ± 1.30	0.979
MUFA	24.87 ± 3.10	23.66 ± 2.88	25.97 ± 2.84^a^	25.77 ± 2.96^b^	23.14 ± 3.35	0.032
Palmitoleic (C16:1)	2.55 ± 0.75	2.41 ± 0.62	2.73 ± .63	2.56 ± 0.89	2.47 ± 0.98	0.658
Oleic (C18:1)	22.32 ± 2.77	21.25 ± 2.54	23.24 ± 2.48^c^	23.22 ± 2.73^d^	20.66 ± 2.89	0.027
PUFA	31.01 ± 4.78	31.65 ± 4.46	30.24 ± 5.37	30.30 ± 3.99	32.94 ± 6.01	0.529
n-6	26.77 ± 4.29	27.26 ± 3.66	25.96 ± 5.13	26.46 ± 3.54	28.26 ± 5.53	0.628
Linoleic (C18:2)	18.06 ± 3.84	18.64 ± 3.29	17.23 ± 4.35	18.10 ± 3.30	18.47 ± 5.39	0.742
Arachidonic (C20:4)	8.71 ± 2.37	8.62 ± 1.73	8.73 ± 2.47	8.36 ± 2.20	9.78 ± 3.89	0.620
n-3	4.23 ± 1.17	4.38 ± 1.56	4.29 ± 1.02	3.84 ± 0.86	4.68 ± 0.91	0.363
a-linolenic (C18:3)	0.54 ± 0.20	0.56 ± 0.20	0.53 ± 0.21	0.52 ± 0.16	0.57 ± 0.31	0.916
EPA (C20:5)	0.46 ± 0.18	0.53 ± 0.24	0.42 ± 0.15	0.43 ± 0.16	0.52 ± 0.11	0.223
DPA (C22:5)	0.83 ± 0.23	0.83 ± 0.21	0.86 ± 0.17	0.80 ± 0.28	0.85 ± 0.33	0.891
DHA (C22:6)	2.39 ± 0.93	2.46 ± 1.26	2.48 ± 0.82	2.10 ± 0.67	2.74 ± 0.73	0.410
n-6/n-3	6.77 ± 2.12	6.80 ± 1.93	6.61 ± 2.96	7.14 ± 1.63	6.16 ± 1.29	0.765
SCD16	0.08 ± 0.02	0.08 ± 0.02	0.09 ± 0.02	0.08 ± 0.02	0.08 ± 0.03	0.398
SCD18	1.85 ± 0.40	1.77 ± 0.38	1.95 ± 0.38	1.90 ± 0.45	1.68 ± 0.36	0.376

Data are presented as mean ± standard deviation. Comparisons were performed by ANOVA way test followed by test t between pairs. *P*-values < 0.05 were considered statistically significant for all statistical test

*EPA*, eicosapentaenoic, *DHA* docosahexaenoic, *DPA* docosapentaenoic *SFA* saturated fatty acid, *MUFA* monounsaturated fatty acid, *PUFA* polyunsaturated fatty acid, *n6* omega-6, *n3* omega-3, *SCD16* stearoyl-CoA-Desaturase 16, SCD18, stearoyl-CoA-Desaturase 18

^a^ *p* = 0.023 for comparison between low lesion vs. without lesion groups by test t

^b^ *p* = 0.040 for comparison between intermediate lesion vs. without lesion groups by test t

^c^ *p* = 0.025 comparison between low lesion vs. without lesion groups by test t

^d^ *p* = 0.034 for comparison between intermediate lesion vs. without lesion groups by test t

**Fig. 1:**
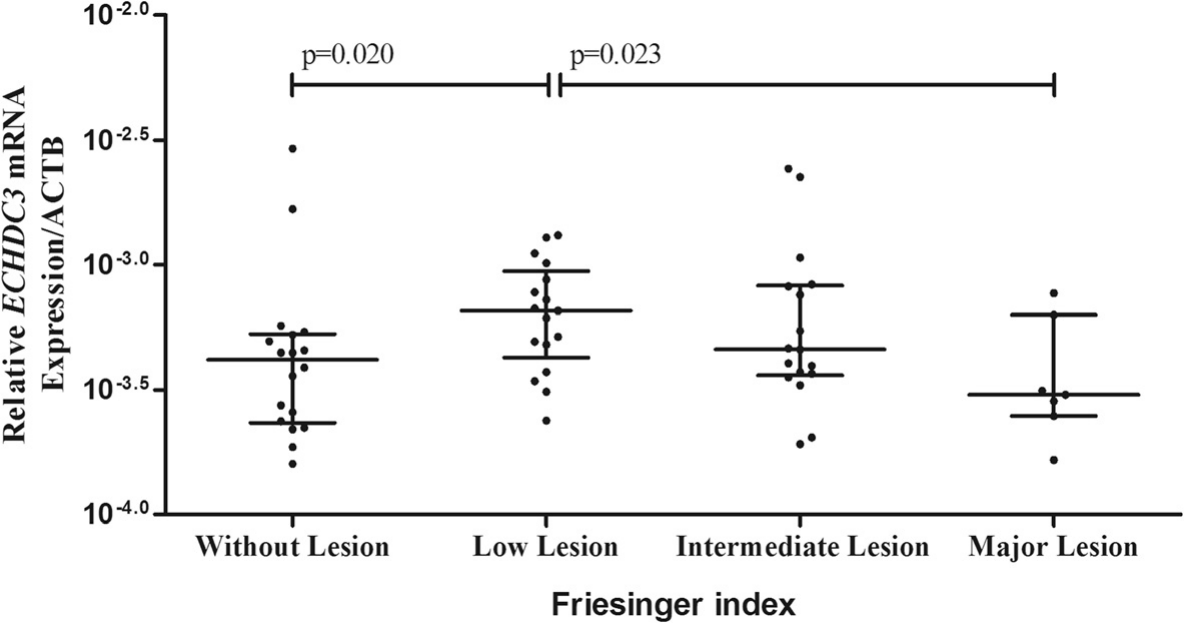
*ECHDC3* mRNA expression relative to *ACTB* expression according to the extent of coronary lesion. Data are presented as median and interquartile range. Relative expression of *ECHDC3* was calculated using the 2-deltaCT method. Statically analysis was performed by Kruskal-Wallis test followed by Mann Whitney test

Regarding mRNA relative expression (Fig. 1), *ECHDC3* mRNA expression were 1.2-fold higher in patients with low lesion than in patients without lesion (*p* = 0.020), and 1.8-fold lower in patients with major lesion than in patients with low lesion (*p* = 0.023).

Pearson analysis showed a positive correlation between oleic acid with triglyceride levels (*r* = 0.397, *p* = 0.002), MUFA (*r* = 0.974, *p* < 0.001), SCD16 (*r* = 0.352, *p* = 0.006) and SCD18 (*r* = 0.751, *p* < 0.001). *ECHDC3* gene expression positively correlated with SFA (*r* = 0.259, *p* = 0.048) and negatively correlated with SCD16 (*r* = −0.289, *p* = 0.027).

## Discussion

In the present study, all patients under CVD risk were classified according to the presence and extent of coronary lesion. Higher levels of MUFA, mainly oleic acid, and *ECHDC3* upregulation were found in patients with low coronary lesion when compared to those without coronary lesion, however we did not find significant difference between patients with major lesions and those without coronary lesion. These findings suggest that both observed high oleic acid levels and *ECHDC3* upregulation may induce initial events of the atherosclerotic process. There is a large number of growth factors, cytokines, regulatory molecules and different cell types that are involved in atherosclerosis development, each having a different function [18]. Furthermore, the mechanisms underlying atherosclerosis differ throughout the stages of disease, and a factor that is important to the initial stage might not be relevant in the late stage, supporting our results.

Oleic acid is an abundant monounsaturated omega-9 fatty acid that affects different biological processes, and is associated with increased expression of genes linked to FA oxidation [19]. These genes are reported to be altered during specific disease conditions that impact the heart, including cardiac failure, myocardial ischemia, and diabetes [20]. In the present study, a positive correlation between oleic acid and triglycerides levels was observed in patients with cardiac lesion, suggesting that there is a potential influence of this FA on the increase of CVD risk. This is supported by a study performed in healthy individuals of both genders showing that virgin olive oil consumption, a oleic acid-rich food, led to upregulation of many genes associated with atherosclerosis disease [21]. Moreover, in cultures of rat aortic smooth muscle cells, the treatment with oleic acid was associated with cell lipid accumulation, increasing foam cell formation in a dose-dependent manner [22].

It was previously reported that *ECHDC3* mRNA expression was significantly increased in patients with ST-segment elevation myocardial infarction when compared to unstable angina, indicating its association with the severity of CVD [3]. The *ECHDC3* role in CVD is scarcely explored to allow a more comparative discussion of our data, but is known that *ECHDC2* upregulation in cardiac tissue of rats was related to increased susceptibility to myocardial ischemia/reperfusion injury [23]. ECHDC3 shares 30 % identity with ECHDC2 [24], and these proteins may possibly have similar functions in the pathophysiology of cardiovascular diseases. In addition, both proteins are named after enoyl-CoA hydratase, an essential enzyme for the β-oxidation of FAs, because they share a conserved domain [7, 23]. The potential involvement of *ECHDC3* in β-oxidation of FA pathway led us to investigate the possible correlation between changes of FA profile and this gene expression.

The high serum levels of oleic acid presently observed might be due to stearoyl-CoA desaturase (SCD) activity, as this fatty acid presented a positive correlation with SCD16 and SCD18 levels. Stearoyl-CoA desaturase (SCD) catalyzes a rate-limiting step in the unsaturated FA synthesis, which main product is oleic acid [25]. Notably, SCD has been associated with inflammatory cluster [16] and metabolic syndrome, reinforcing the impression of a detrimental association of oleic acid levels and CVD risk [26].

We do recognize some limitations of our study: the diet of individuals was not studied, and the sample size is small. However, to the best our knowledge, it is the first study evaluating the relationship between *ECHDC3* mRNA expression and FA levels in patients classified according to the Friesinger index.

## Conclusion

Our data suggests that increased oleic acid levels and ECHDC3 upregulation in individuals with coronary artery lesion could be independent factors associated to the early CVD development. More investigation is needed to validate this hypothesis and to consider whether the monitoring of FA serum levels, especially the oleic acid, would be valuable for the assessment of cardiovascular risk.

## Abbreviations

CVD: Cardiovascular disease
DHA: Docosahexaenoic
DPA: Docosapentaenoic
EPA: Eicosapentaenoic
FA: Fatty acid
MUFA: Monounsaturated fatty acid
n3: Omega-3
n6: Omega-6
PUFA: Polyunsaturated fatty acid
SFA: Saturated fatty acid.
